# Dopamine D2-receptor neurons in nucleus accumbens regulate sevoflurane anesthesia in mice

**DOI:** 10.3389/fnmol.2023.1287160

**Published:** 2023-11-28

**Authors:** Li Niu, Mengnan Hao, Yanhong Wang, Kai Wu, Chengdong Yuan, Yu Zhang, Jie Zhang, Xiaoli Liang, Yi Zhang

**Affiliations:** ^1^Department of Anesthesiology, The Second Affiliated Hospital of Zunyi Medical University, Zunyi, China; ^2^Guizhou Key Laboratory of Anesthesia and Organ Protection, Zunyi Medical University, Zunyi, China; ^3^School of Anesthesiology, Zunyi Medical University, Zunyi, China; ^4^Department of Anesthesiology, The Affiliated Hospital of Zunyi Medical University, Zunyi, China

**Keywords:** general anesthesia, neural pathway, sevoflurane, nucleus accumbens (NAc), acceptor

## Abstract

**Introduction:**

The mechanism of general anesthesia remains elusive. In recent years, numerous investigations have indicated that its mode of action is closely associated with the sleep-wake pathway. As a result, this study aimed to explore the involvement of dopamine D2 receptor (D2R) expressing neurons located in the nucleus accumbens (NAc), a critical nucleus governing sleep-wake regulation, in sevoflurane anesthesia.

**Methods:**

This exploration was carried out using calcium fiber photometry and optogenetics technology, while utilizing cortical electroencephalogram (EEG), loss of righting reflex (LORR), and recovery of righting reflex (RORR) as experimental indicators.

**Results:**

The findings from calcium fiber photometry revealed a decrease in the activity of NAc^D2R^ neurons during the induction phase of sevoflurane anesthesia, with subsequent recovery observed during the anesthesia’s emergence phase. Moreover, the activation of NAc^D2R^ neurons through optogenetics technology led to a reduction in the anesthesia induction process and an extension of the arousal process in mice. Conversely, the inhibition of these neurons resulted in the opposite effect. Furthermore, the activation of NAc^D2R^ neurons projecting into the ventral pallidum (VP) via optogenetics demonstrated a shortened induction time for mice under sevoflurane anesthesia.

**Discussion:**

In conclusion, our research outcomes suggest that NAc^D2R^ neurons play a promotive role in the sevoflurane general anesthesia process in mice, and their activation can reduce the induction time of anesthesia via the ventral pallidum (VP).

## 1 Introduction

The mechanism underlying the alterations in consciousness induced by general anesthesia has long remained an enigmatic puzzle. In recent times, as research in related fields has deepened, it has been discovered that the manifestations of consciousness loss and reduced muscle tone induced by general anesthetics bear a striking resemblance to the characteristics exhibited during natural sleep processes (van der Meij et al., 2019). Remarkably, there are also noteworthy similarities between the two states of unconsciousness, (Franks, 2008) and an increasing body of evidence suggests that general anesthesia might share common neural pathways and action targets with the sleep-wake cycle (Zhang et al., 2019; Luo et al., 2020). Consequently, investigating the mechanism of general anesthesia through the lens of the sleep-wake pathway has emerged as a prominent topic in the fundamental research of anesthesiology in recent years.

The nucleus accumbens (NAc), situated in the ventral striatum, constitutes a significant structure within the basal forebrain. Serving as a principal constituent of the midbrain limbic dopaminergic system, the nucleus accumbens maintains intimate fiber connections with various sleep-wake structures and is considered a pivotal nucleus for consciousness regulation within the brain (Li et al., 2018; Monti and Jantos, 2018; Gretenkord et al., 2020; Gui et al., 2021). In an earlier study involving rats, Tellez et al. (2012) found that the majority of neurons in the NAc exhibited a strong inhibitory effect during slow-wave sleep. Research findings have indicated that the nucleus accumbens (NAc) serves as the primary site for dopamine receptor accumulation in the brain, encompassing approximately 95% of medium spiny GABAergic projection neurons (MSNs) (Castro and Bruchas, 2019). Based on the distinct receptor expression, these neurons can be categorized into subtypes that express dopamine D1 receptors (D1-MSNs) and dopamine D2 receptors (D2-MSNs) (Scofield et al., 2016). Certain researchers have observed that the activation of NAc^D1*R*^ neurons can rapidly transition mice from non-rapid eye movement sleep (NREMs) to a state of wakefulness, whereas inhibiting these neurons leads to the opposite effect (Luo Y. J. et al., 2018). Moreover, during anesthesia, the activation of NAc^D1*R*^ neurons prolongs the induction process while shortening the recovery process in mice (Zhang et al., 2023). These findings strongly indicate the pivotal role of NAc^D1*R*^ neurons in the regulation of consciousness during both sleep and anesthesia.

Nonetheless, there remains a paucity of reports concerning the potential involvement of NAc^D2R^ neurons in consciousness regulation. In a study focusing on sleep regulation, researchers noted that the inhibition of NAc^D2R^ neurons heightened the arousal of mice and notably diminished both non-rapid eye movement sleep (NREMS) and rapid eye movement sleep (REMs) (Luo Y. J. et al., 2018). This observation suggests that NAc^D2R^ neurons may serve as a crucial element in maintaining sleep and regulating the reduction of consciousness activity. However, the precise role of NAc^D2R^ neurons in the process of general anesthesia and its specific mechanisms still remain unclear.

As a pivotal brain nucleus involved in consciousness regulation, the nucleus accumbens (NAc) exhibits close associations with multiple wake/sleep structures, such as the ventral tegmental area (VTA), substantia nigra (SN), ventrolateral preoptic nucleus (VLPO), and ventral pallidum (VP), (Salgado and Kaplitt, 2015; Mingote et al., 2019) with VP being particularly noteworthy. The VP is located in the ventral portion of the anterior commissure, akin to the NAc, and approximately 80% of its composition comprises GABAergic neurons (Root et al., 2015). Serving as a prominent output core of the basal forebrain, the VP predominantly receives neuronal innervation from the NAc and projects inhibitory projections to various brain regions (Kupchik et al., 2015; Root et al., 2015; Pardo-Garcia et al., 2019). In a study by Oishi et al. (2017) optogenetic activation of NAc neuronal terminals within the VP was found to decrease the firing rate of VP neurons and induce inhibitory postsynaptic currents, a phenomenon not observed in other downstream nuclei of the NAc. Furthermore, Bao et al. (2023) reported that the activation of the NAc^D1*R*^-VP pathway could facilitate the recovery of mice from sevoflurane anesthesia. These studies collectively demonstrate the close relationship between the NAc and VP, as well as their ability to regulate the process of general anesthesia. However, the role of the NAc^D2R^-VP pathway warrants further investigation.

Thus, in this investigation, we employed D2R-Cre mice as the experimental animal model to examine the regulatory function of NAc^D2R^ neurons in sevoflurane anesthesia, utilizing calcium fiber photometry and optogenetics as research tools. The experimental outcomes unveiled the crucial role of NAc^D2R^ neurons and the NAc^D2R^-VP pathway in governing sevoflurane general anesthesia, thereby establishing a fundamental theoretical foundation for the exploration of the mechanisms underlying general anesthesia.

## 2 Materials and methods

### 2.1 Animals

In this experiment, healthy adult male D2R-Cre mice (8–10 weeks of age) with a weight range of 20–25 g were initially included, totaling 54 mice at the commencement of the study. However, due to modeling and experimental mishaps, five mice succumbed, and subsequently, this portion of the sample size was replenished. All mice were housed in the specific pathogen-free (SPF) standard facility at the Zunyi Medical University Experimental Animal Center. These mice were provided unrestricted movement and access to food. The animal facility adhered to a circadian rhythm for the mice, with 12 h of light (20:00–8:00) and 12 h of darkness (8:00–20:00), maintaining a room temperature of 23 ± 2 °C and a relative humidity of 55 ± 5%. All experimental procedures were conducted in accordance with the guidelines set forth by the Animal Care and Use Committee of Zunyi Medical University.

### 2.2 Drugs

Sevoflurane was acquired from RWD Life Science, the vendor based in Shenzhen, China. The dopamine D2R/DRD2 antibody (55084-1-AP) was purchased from Proteintech Group, Inc., located in the United States. As for the secondary antibodies, we used goat anti-rabbit antibodies conjugated to Alexa 594 (ab150080, Abcam, Shanghai, China).

### 2.3 Virus injection

The mice underwent anesthesia with a mixture of 1.4% isoflurane and oxygen (1.5 l/min). Once the righting reflex disappeared and they no longer responded to pain stimulation, the mice were immobilized on the stereotactic instrument (RWD Life Science). To protect their eyes from strong light injury, erythromycin ointment was applied, and cotton balls were used to cover their eyes. The operation area was disinfected with iodophor, and subcutaneous injection of lidocaine (1%) provided local anesthesia. After the local anesthesia took effect, a skin incision was made, and the periosteum was removed to expose the skull seam. Head leveling was performed according to the bregma and lamda points. Based on the third edition of the mouse brain atlas, the injection sites for NAc (anterior–posterior [AP]: +1.5 mm, medial-lateral [ML]: +0.7 mm, and dorsal-ventral [DV]: -4.5 mm) and VP (AP: +0.5 mm, ML: +1.5 mm, DV: −5.1 mm) were determined and labeled. A small opening with a diameter of 300–500 μm was created above the point of virus injection and optical fiber implantation using a skull drill. The following Adeno-associated viruses were injected at a rate of 12 nl/min through the microinjection pump and left for 10 min after injection: rAAV-hSyn-DIO-GCaMP6s-WPRE-pA, rAAV-Eflα-DIO-hChR2-EYFP-WPRE-pA, AAV-Eflα-DIO-eNpHR-EYFP-WPRE-pA, rAAV-Eflα-EYFP-WPRE-pA. Optical fibers were embedded 0.3 mm above the virus area.

In the optogenetic experiment, we used six-channel wire-style cortical electroencephalogram (EEG) electrodes (KD-EEG, KedouBC), and the installation procedure was as follows: four skull holes were selected with bregma as the reference point, at the coordinates anterior-posterior: +1 mm, medial-lateral: ± 1.5 mm, and anterior-posterior: −3.5 mm, medial-lateral: ± 1 mm, respectively. The tips of the skull screws were implanted into the skull to make direct contact with the brain cortex, and the head ends of the skull screws were connected to the four silver wires leading from the electrodes. Finally, dental cement was used to secure the skull screws and electrodes on the top of the mice’s heads, facilitating the conduction of electrical signals from the skull screws to the electrodes and the subsequent signal collection process.

### 2.4 Behavioral test

Loss of righting reflex (LORR) and recovery of righting reflex (RORR) in mice are widely regarded as standardized indicators of general anesthesia induction and recovery time. Prior to commencing the experiment, the mice were placed in an induction box (10 cm × 20 cm × 15 cm) for 10 min. Subsequently, the mice were induced and maintained with 2.4% sevoflurane mixed with 100% oxygen at a flow rate of 1.5 L/min. Throughout the entire procedure, the concentration of sevoflurane in the anesthesia room was monitored using an anesthesia monitor from Drager Company. The time from the initiation of sevoflurane administration to the loss of righting reflex was recorded as LORR time, whereas the time from the cessation of sevoflurane administration to the recovery of righting reflex was recorded as RORR time.

### 2.5 Calcium fiber photometry

The changes in Ca^2+^ signals in mice were captured using a fiber photometry system (ThinkerTech Nanjing Bioscience, Nanjing, Jiangsu, China). This system is equipped with a 480 nm excitation LED (3W, CREE), a dichroic mirror (DCC3420M; Thorlabs, Shanghai, China), and a multifunctional data collection program (ThinkerTech Nanjing Bioscience Inc.). To facilitate light transmission between the system and the implanted optical fiber, an optical fiber produced by Newton company in China and an optical splitter from Doric Lenses company were utilized. The Ca^2+^ signals of mice in the awake state were recorded as the baseline fluorescence intensity and denoted as F0. F represents the test fluorescence intensity, expressed as (F-F0)/F0 = ΔF/F, indicating the fluorescence intensity changes in response to the stimulus. The acquired data were subjected to analysis using MATLAB 2016a (MathWorks, Cambridge, United States).

### 2.6 Optogenetics

Optogenetics alow genetically manipulation of nerve cells expressing photosensitive ion channels, enabling precise regulation of the activity of target neurons. Before the experiment, the mice were acclimatized in the induction box for 10 min, and their awake electroencephalogram (EEG) was recorded for 5 min. Anesthesia induction was achieved using a mixture of 2.4% sevoflurane with oxygen (1.5 L/min). Concurrently, the optogenetic system activated (473 nm) or inhibited (589 nm) the neurons or nerve terminals until the mice displayed loss of righting reflex (LORR), at which point the light supply was halted. The anesthesia was maintained with a sevoflurane concentration of 2.4% for 20 min. Toward the end of anesthesia, the optogenetic system was once again utilized to activate or inhibit neurons until the recovery of righting reflex (RORR) was observed, and light stimulation was ceased. EEG was recorded for 5 min during resuscitation. Standardized light stimulation parameters were employed, including a frequency of 20 Hz and a pulse width of 10 ms. The power of light was calibrated between 10 and 15 milliwatts. Subsequently, the EEG data were imported into Spike2 software for analysis.

### 2.7 Histological verification

Under isoflurane inhalation (1.4%), the mice were transcranially injected with 150 ml of PBS and 50 ml of 4% paraformaldehyde (PFA). The brains were then removed and immersed in 4% PFA for 24 h at 4°C in cold storage. Subsequently, the brains were transferred to 30% sucrose PBS at the same temperature until they sank. After dehydration, brain sections were prepared using a freezing microtome with a thickness of 30 μm. Brain slices containing the target brain regions were placed in a blocking solution and blocked for 1 h at room temperature, which consisted of 2.5% goat serum, 1.5% bovine serum albumin, and 0.1% TritonTM X-100. Following this, the cells were incubated with a primary antibody (rabbit anti-DRD2 antibody) diluted to 1:200 in the blocking solution for 12 h at 4°C. The brain slices were then washed three times with PBS for 10 min each time. Subsequently, the slices were further treated with a secondary antibody (goat anti-rabbit antibody) combined with Alexa 594 at a dilution of 1:1,000 for 2 h at room temperature and washed with PBS. The slices were mounted on glass slides and coated with mounting material (Gold antifade reagent with DAPI, Life Technologies, Beijing, China). Finally, immunostaining images were acquired using the Olympus BX63 Virtual Microscope System.

### 2.8 Statistical analysis

Statistical analysis was performed using GraphPad Prism 8.0 software package (GraphPad Software Inc, Beijing, China). The normality of data distribution was assessed using the Shapiro–Wilk test. To compare the changes in calcium fiber photometry events before and after, a repeated measures one-way analysis of variance (ANOVA) was employed. For comparing the changes in LORR and RORR time in the optogenetic experiments, a repeated measures two-way ANOVA was used. To compare the percentage of EEG frequency bands between different groups, independent samples *t*-tests were utilized. In all analyses, a significance level of *p* < 0.05 was considered statistically significant.

## 3 Results

### 3.1 Sevoflurane anesthesia inhibits the activity of NAc^D2R^ neurons

**FIGURE 1:**
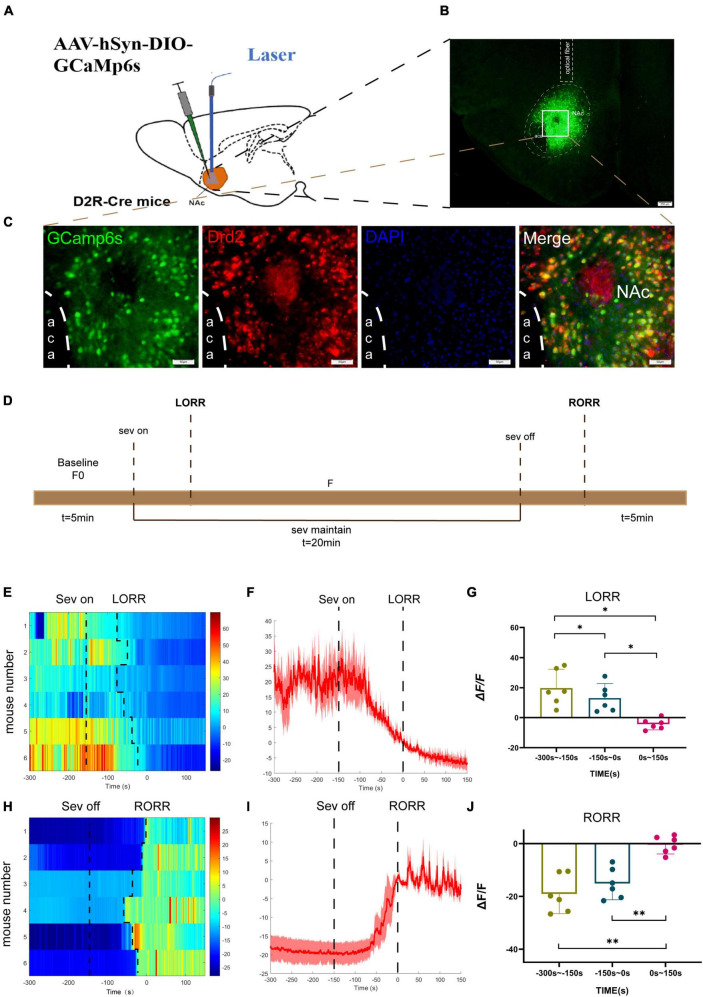
Phase- dependent calcium alteration in NAc^D2R^ neurons during sevoflurane anesthesia. **(A)** Schematic diagram of establishing calcium signal recording model in D2R CRE mice; **(B)** virus injection and optical fiber embedding position (scale = 200 μm). **(C)** Expression of GCaMP6s in NAc^D2R^ neurons. Viral expression (GCaMP6s, green) in the NAc and colabeling with D2R expressing neurons (Drd2 immunofluorescence, red; DAPI, blue; scale: 50 mm). **(D)** Schematic diagram of fiber photometric experiment flow. **(E,H)** Thermogram of calcium signal changes in LORR and RORR phases during sevoflurane anesthesia. **(F,I)** Average calcium transients associated with LORR and RORR. **(G, J)** ΔF/F is the deviation of Ca^2+^ fluorescence from baseline. The data is presented as a 95% confidence interval. *, *p* < 0.05; **, *p* < 0.01; *n* = 6, repeated measures one-way analysis of variance, RM-ANOVA.

To investigate the real-time activity of NAc^D2R^ neurons during sevoflurane anesthesia, we conducted experiments using Cre-dependent adeno-associated virus (AAV) containing the fluorescent calcium indicator GCaMP6s, which was injected into the NAc of D2R-Cre mice (Figure 1B). Fiber photometry was then utilized to record the changes in Ca2+ signals in mice during sevoflurane anesthesia. Immunofluorescence staining confirmed the efficient and specific transfection of the virus into D2R neurons (Figures 1A–D).

During the induction phase of sevoflurane anesthesia, we divided the analysis of Ca2+ signals into three time periods: the wake-up period (−300 to −150 s), the induction period (−150 to 0 s), and the anesthesia period (0 to 150 s). In the recovery period of sevoflurane anesthesia, we analyzed three stages, including the anesthesia period (−300 to −150 s), the recovery period (−150 to 0 s), and the wake-up period (0 to 150 s).

The experimental findings demonstrated that the calcium signal of NAc^D2R^ neurons during sevoflurane anesthesia induction (*p* < 0.05) and sevoflurane anesthesia (*p* < 0.05) was significantly lower than during the awake period (Figures 1E–G). In contrast, the calcium signal of NAc^D2R^ neurons increased significantly during the recovery and awake periods (*p* < 0.01) when compared to the anesthesia period (Figures 1H–J).

In summary, our results indicate that the activity of NAc^D2R^ neurons decreases during sevoflurane anesthesia induction and increases during the recovery phase, suggesting that sevoflurane anesthesia can inhibit the activity of NAc^D2R^ neurons.

### 3.2 Optogenetic activation of NAc^D2R^ neurons was found to facilitate the induction process of sevoflurane anesthesia and extend the awakening process

**FIGURE 2:**
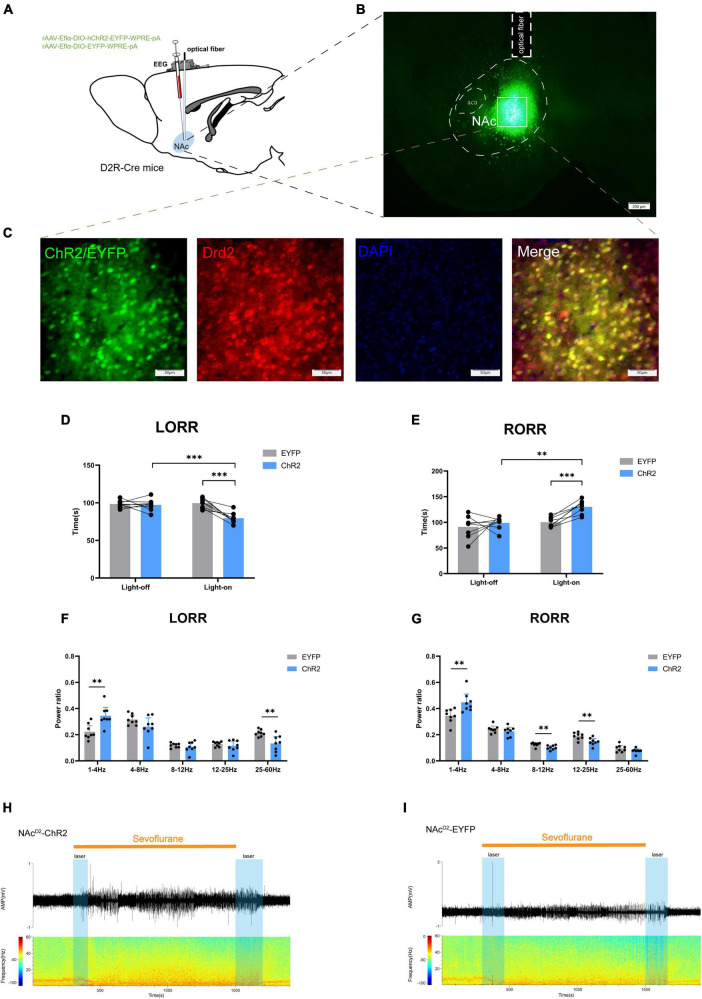
Optogenetic activation of NAc^D2R^ neurons promotes sevoflurane anesthesia. **(A)** Schematic diagram of optogenetic model establishment; **(B)** virus injection and optical fiber embedding position (scale = 200 μm); **(C)** green represents virus (ChR2/EYFP), red represents NAc^D2R^ neurons, blue represents DAPI, and yellow represents NAc^D2R^ neurons transfected with virus (scale = 50 μm); **(D)** Optogenetic activation of NAc^D2R^ neurons shortened the LORR time of mice under 2.4% sevoflurane anesthesia (ChR2 vs. EYFP, *P* < 0.001; ChR2-on vs. ChR2-off, *P* < 0.001); **(F)** light activated NAc^D2R^ neurons during LORR lead to δ bands increased (*P* < 0.01), γ bands reduction (*P* < 0.01); **(E)** optogenetic activation of NAc^D2R^ neurons prolonged the RORR time of mice under 2.4% sevoflurane anesthesia (ChR2 vs. EYFP, *P* < 0.001; ChR2-on vs. ChR2-off, *P* < 0.01); **(G)** light activated of NAc^D2R^ neurons during RORR leads to δ bands increase (*P* < 0.01), β bands and α bands reduction (*P* < 0.01); **(H,I)** representative electroencephalogram during LORR and RORR. The data is presented as a 95% confidence interval. **, *P* < 0.01; ***, *P* < 0.001; *n* = 8.

In order to investigate the regulatory role of NAc^D2R^ neurons in sevoflurane anesthesia, we injected rAAV-Eflα-DIO-hChR2-EYFP-WPRE-pA and rAAV-Eflα-DIO-EYFP-WPRE-pA into the NAc of D2R-Cre mice (Figure 2B). Immunofluorescence staining confirmed the efficient and specific labeling of NAc^D2R^ neurons by the virus (Figures 2A–C).

The behavioral analysis revealed significant differences between the EYFP group and the light-activated NAc^D2R^ neurons (ChR2) group during sevoflurane anesthesia. Specifically, activating NAc^D2R^ neurons shortened the loss of righting reflex (LORR) time in mice [10.59, 29.66] (*p* < 0.0001) and prolonged the recovery of righting reflex (RORR) time [−50.49, −8.507] (*p* < 0.01) compared to the EYFP group (Figures 2D, E). Moreover, when comparing to the self-control group without light, the activation of NAc^D2R^ neurons significantly reduced the LORR time [8.217, 27.28] (*p* < 0.0001) and extended the RORR time [−52.12, −10.13] (*p* < 0.01) (Figures 2D, E).

Cortical EEG data showed that during sevoflurane anesthesia induction, the ChR2 group exhibited an increase in δ waves (1–4 Hz, *p* < 0.05) and a decrease in γ waves (25–60 Hz, *p* < 0.01). In the recovery period of sevoflurane anesthesia, the ChR2 group demonstrated an increase in δ waves (*p* < 0.01) and a reduction in β waves (12–25 Hz) and α waves (8–12 Hz) (Figures 2F–I). These results indicate that throughout the LORR and RORR phases, the application of blue light to the ChR2 group resulted in amplified low-frequency wave bands and attenuated high-frequency wave bands. This suggests that the activation of NAc^D2R^ neurons plays a regulatory role in both the induction and awakening processes of sevoflurane anesthesia.

### 3.3 Optogenetic inhibition of NAc^D2R^ neurons was found to delay sevoflurane induction and accelerate awakening

**FIGURE 3:**
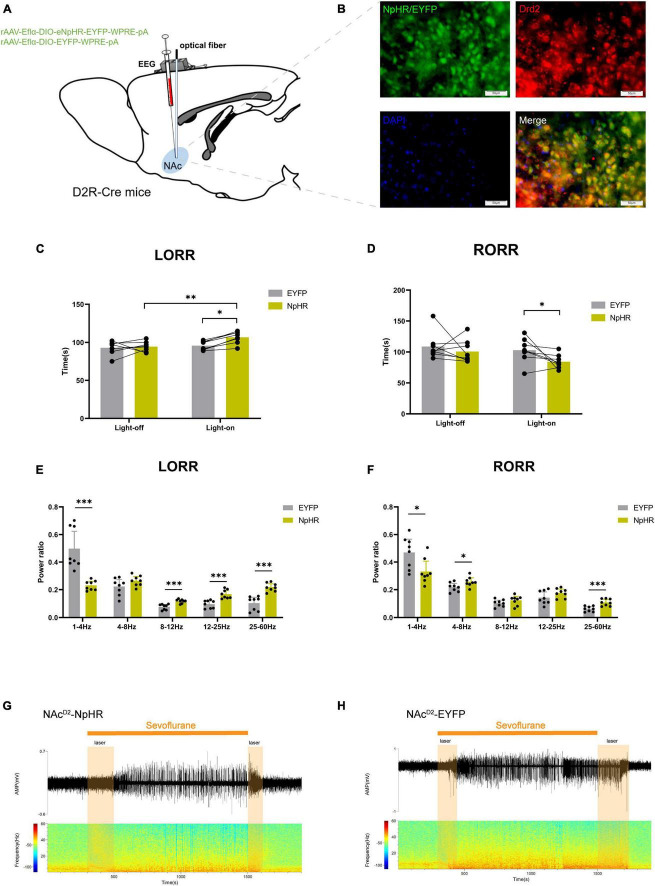
Optogenetic inhibition of NAc^D2R^ neurons delays sevoflurane anesthesia. **(A)** Schematic diagram of optogenetic model establishment; **(B)** green represents virus (NpHR/EYFP), red represents NAc^D2R^ neurons, blue represents DAPI, and yellow represents NAc^D2R^ neurons transfected with virus (scale = 50 μm); **(C)** optogenetic inhibition of NAc^D2R^ neurons prolonged the LORR time of mice under 2.4% sevoflurane anesthesia (NpHR vs. EYFP, *P* < 0.05; NpHR-on vs. NpHR-off, *P* < 0.01); **(E)** inhibition of NAc^D2R^ neurons during LORR resulted in δ bands reduction (*P* < 0.001), β, α and γ bands increase (*P* < 0.001); **(D)** optogenetic inhibition of NAc^D2R^ neurons shortened the RORR time of mice under 2.4% sevoflurane anesthesia (NpHR vs. EYFP, *P* < 0.05); **(F)** inhibition of NAc^D2R^ neurons during RORR resulted in δ bands reduction (*P* < 0.05), θ bands (*P* < 0.05) and γ bands increase (*P* < 0.001); **(G,H)** representative electroencephalogram during LORR and RORR. The data is presented as a 95% confidence interval. *, *P* < 0.05; **, *P* < 0.01; ***, *P* < 0.001; *n* = 8.

Subsequently, we injected rAAV-Eflα-DIO-eNPHR-EYFP-WPRE-pA and rAAV-Eflα-DIO-EYFP-WPRE-pA into the NAc of D2R-Cre mice (Figure 3A). Immunofluorescence staining confirmed the effective and specific labeling of NAcD2R neurons by the virus (Figure 3B).

Compared to the EYFP group, the optogenetic inhibition of NAc^D2R^ neurons led to a prolonged loss of righting reflex (LORR) time [−20.83, −1.170] (*p* < 0.05) and shortened the recovery of righting reflex (RORR) time [0.1681, 37.08] (*p* < 0.05) during sevoflurane anesthesia in mice. Additionally, when compared to the control group without light, the inhibition of NAc^D2R^ neurons prolonged the LORR time [−22.20, −2.545] (*p* < 0.01) (Figures 3C, D). Cortical EEG analysis showed that during sevoflurane anesthesia induction, the NpHR group exhibited a reduction in δ waves (*p* < 0.001) and an increase in α, β, and γ waves amplitude (*p* < 0.001). In the recovery period of sevoflurane anesthesia, the NpHR group demonstrated a decrease in δ waves (*p* < 0.05) and an increase in θ waves (4–8 Hz) and γ waves (*p* < 0.001) (Figures 3E–H).

Based on the above findings, it has been observed that the activation of NAc^D2R^ neurons can enhance sevoflurane anesthesia in mice. Conversely, the inhibition of NAc^D2R^ neurons can elevate the consciousness level in mice and render them less susceptible to anesthesia.

### 3.4 Optogenetic activation of NAc^D2R^-VP pathway accelerates sevoflurane induction

**FIGURE 4:**
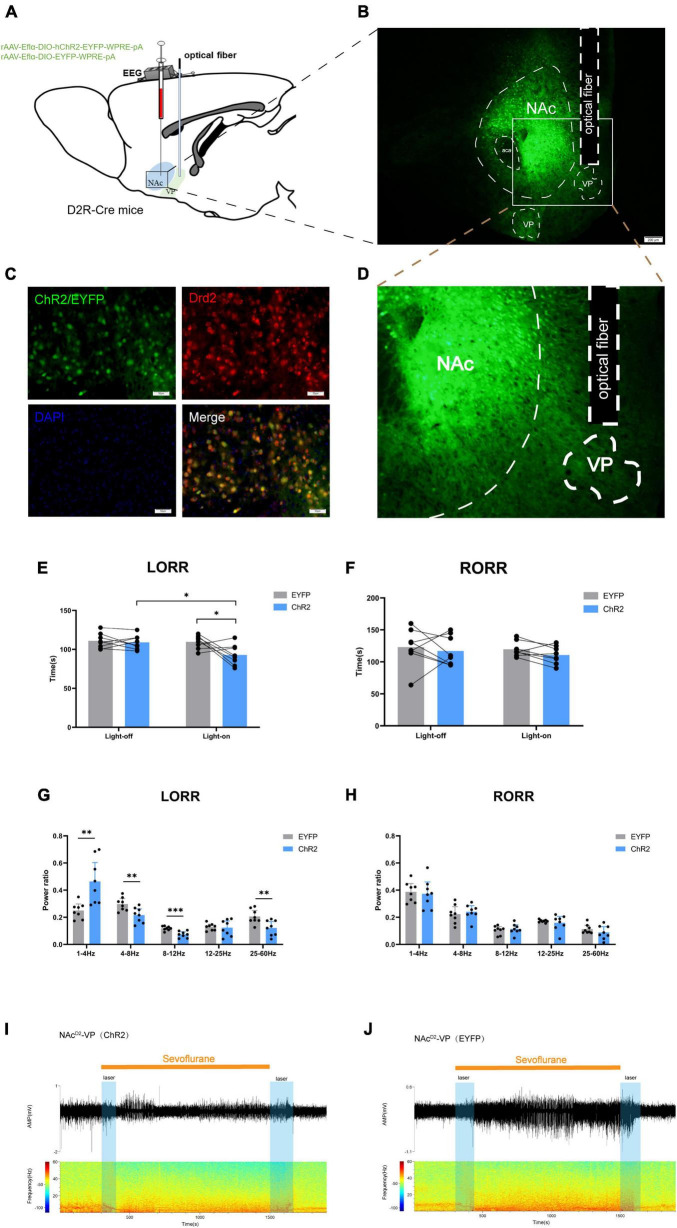
Optogenetic activation of NAc^D2*R*^-VP pathway promotes sevoflurane induction. **(A)** Schematic diagram of optogenetic model establishment; **(B)** virus injection and optical fiber embedding position (scale = 200 μm); **(D)** Expression of NAc^D2R^ neuronal terminals in VP; **(C)** green represents virus (ChR2/EYFP), red represents NAc^D2R^ neurons, blue represents DAPI, and yellow represents NAc^D2R^ neurons transfected with virus (scale = 50 μm); **(E)** optogenetic activation of NAc^D2R^ -VP pathway shortened the LORR time of mice under 2.4% sevoflurane anesthesia (ChR2 vs. EYFP, *P* < 0.05; ChR2-on vs. ChR2-off, *P* < 0.05); **(G)** activation of NAc^D2R^ -VP pathway during LORR resulted in δ bands increased (*P* < 0.01), α bands reduction (*P* < 0.001), and θ, γ bands reduction (*P* < 0.01); **(F)** effect of optogenetic activation of NAc^D2R^ -VP pathway on RORR time in mice; **(H)** proportion of brain wave frequency in mice with light activated NAc^D2R^ -VP pathway during RORR; **(I,J)** representative electroencephalogram during LORR and RORR. The data is presented as a 95% confidence interval. *, *P* < 0.05; **, *P* < 0.01; ***, *P* < 0.001; *n* = 8.

The study aimed to elucidate the pathway through which NAc^D2R^ neurons exert their regulatory effects on general anesthesia. Given the close and unique relationship between NAc and VP, we opted to manipulate the NAc^D2R^–VP pathway to identify the target of NAc^D2R^ neurons in regulating general anesthesia. To accomplish this, we performed an integration of optogenetic virus (rAAV-Eflα-DIO-hChR2-EYFP-WPRE-pA and rAAV-Eflα-DIO-EYFP-WPRE-pA) injection into the NAc of D2R-Cre mice, and subsequently implanted optical fibers in the VP (Figures 4A–D). By activating or inhibiting the axon terminals of NAc^D2R^ neurons in the VP, we observed the changes in pathway activity and its impact on sevoflurane anesthesia.

The findings indicated that optogenetic activation of the NAc^D2R^-VP pathway, when compared to the EYFP group, significantly reduced the loss of righting reflex (LORR) time in mice under sevoflurane anesthesia [2.748, 30.75] (*p* < 0.05). Furthermore, this activation resulted in an increase in δ waves (*p* < 0.01) and a decrease in θ waves (*p* < 0.01), α waves (*p* < 0.001), and γ waves (*p* < 0.01) during sevoflurane anesthesia (Figures 4E, G, I, J). However, no significant effect on the recovery of righting reflex (RORR) time was observed (Figures 4F, H). Additionally, when compared to the control group without light, optogenetic activation of the NAc^D2R^-VP pathway also led to a shortened LORR time [2.123, 30.13] (*p* < 0.05) without significantly affecting the RORR time (Figures 4E, F).

### 3.5 Optogenetic inhibition of NAc^D2R^-VP pathway delays sevoflurane induction

**FIGURE 5:**
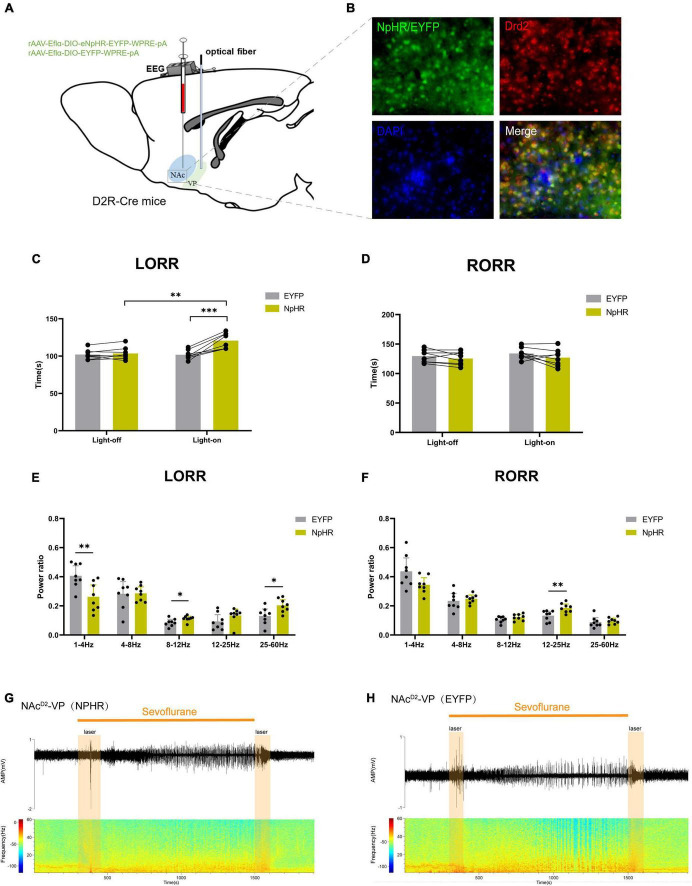
Optogenetic inhibition of NAc^D2R^ -VP pathway delays sevoflurane anesthesia induction. **(A)** Schematic diagram of optogenetic model establishment; **(B)** green represents virus (NpHR/EYFP), red represents NAc^D2R^ neurons, blue represents DAPI, and yellow represents NAc^D2R^ neurons transfected with virus (scale = 50 μm); **(C)** optogenetic inhibition of the NAc^D2R^ -VP pathway prolonged the LORR time of mice under 2.4% sevoflurane anesthesia (NpHR vs. EYFP, *P* < 0.001; NpHR-on vs. NpHR-off, *P* < 0.01); **(E)** inhibition of the NAc^D2R^ -VP pathway during LORR resulted in δ bands reduction (*P* < 0.01), α bands and γ bands increase (*P* < 0.05); **(D)** effect of optogenetic inhibition of NAc^D2R^ -VP pathway on RORR time in mice; **(F)** Proportion of brain wave frequency in mice with inhibition of NAc^D2R^ -VP pathway during RORR; **(G,H)** Representative electroencephalogram during LORR and RORR. The data is presented as a 95% confidence interval. *, *P* < 0.05; **, *P* < 0.01; ***, *P* < 0.001; *n* = 8.

In order to further explore the role of the NAc^D2R^-VP pathway during the sevoflurane general anesthesia process, we injected rAAV-Eflα-DIO-eNPHR-EYFP-WPRE-pA and rAAV-Eflα-DIO-EYFP-WPRE-pA into the NAc of D2R-Cre mice (Figure 5A). Immunofluorescence staining confirmed the effective and specific labeling of NAc^D2R^ neurons by the virus (Figure 5B).

The data demonstrated that inhibition of the NAc^D2R^-VP pathway, in comparison to the EYFP group, significantly prolonged the loss of righting reflex (LORR) time during sevoflurane anesthesia in mice [−29.82, −7.926] (*p* < 0.001). Furthermore, cortical EEG analysis revealed a reduction in δ waves (*p* < 0.01) and an increase in α waves and γ waves (*p* < 0.05) (Figures 5C, E, G, H), while no significant effect was observed on the recovery of righting reflex (RORR) time (Figures 5D, F). Similarly, when compared to the control group without light, optogenetic stimulation of the NAc^D2*R*^-VP pathway using yellow light also resulted in a prolonged LORR time [−28.20, −6.301] (*p* < 0.01), with no significant impact on the RORR time (Figures 5C, D).

These findings suggest that modulating the activity of the NAc^D2*R*^-VP pathway can effectively regulate the induction process of sevoflurane anesthesia in mice. However, there appears to be no significant alteration in the recovery process.

## 4 Discussion

The objective of this study was to investigate the involvement of NAc^D2R^ neurons and the NAc^D2*R*^-VP pathway in sevoflurane anesthesia. The findings revealed that optogenetic activation of NAc^D2R^ neurons and the NAc^D2*R*^-VP pathway led to the acceleration of righting reflex extinction and cortical EEG depression in mice during sevoflurane anesthesia. These results provide evidence that NAc^D2R^ neurons play a crucial role in promoting the process of sevoflurane anesthesia and contribute to the accelerated induction of anesthesia through targeted regulation of VP.

Calcium fiber photometry, being one of the most precise and rapid techniques for monitoring neuronal activity, enables real-time assessment of dynamic changes in neuronal function. In our study, we observed a decrease in the activity of NAc^D2R^ neurons during sevoflurane anesthesia, which aligns with previous research indicating that commonly used anesthetic drugs can lead to the overall suppression of neural activity in the brain (Fosque et al., 2015; Awal et al., 2018, 2020). Our findings further support the notion that anesthesia induces a generalized inhibition of neuronal function (Luo T. et al., 2018; Luo et al., 2020; Xu et al., 2020; Bao et al., 2021).

To elucidate the role of NAc^D2R^ neurons in sevoflurane anesthesia and validate our hypothesis, we employed optogenetics to manipulate NAc^D2R^ neurons. The outcomes demonstrated that specific activation of NAc^D2R^ neurons expedited the induction phase and extended the recovery phase during sevoflurane anesthesia in mice. Additionally, it led to an increase in δ wave power and a reduction in β and γ wave power. Conversely, inhibition of NAc^D2R^ neurons yielded opposite effects. Interestingly, these findings contrast with previous reports on the regulation of general anesthesia by NAc^D1R^ neurons. Our results shed light on the distinct roles of NAc^D2R^ neurons in the anesthesia process, providing valuable insights into the complexity of anesthesia modulation in the brain.

For instance, a study conducted by Bao et al. (2021) utilized chemogenetic methods to activate NAc^D1R^ neurons, leading to a delay in the induction of sevoflurane anesthesia. We hypothesize that this outcome might arise from the distinct projection areas of these two neuron populations. Previous research has demonstrated that D1-MSNs in the NAc directly project to the midbrain region (direct pathway), whereas D2-MSNs indirectly project to the midbrain region via the globus pallidus and subthalamic nucleus (indirect pathway) (Bock et al., 2013). The activation of dopamine receptors on each MSN subtype triggers different intracellular signaling cascades. Specifically, D2 receptor activation inhibits PKA activity through Gi signals in D2-MSNs, whereas D1 receptor activation stimulates PKA activity through Gs/olf signals in D1-MSNs (Lobo and Nestler, 2011). These two MSN subtypes and their parallel pathways usually exert complementary or even opposing effects on behavior controlled by the cortical striatum system, (Gerfen and Surmeier, 2011) which may explain why these two neurons play contrasting roles in regulating the process of general anesthesia.

While our findings indicate the involvement of NAc^D2R^ neurons in the regulation of general anesthesia, the downstream targets and effect pathways through which NAc^D2R^ neurons exert their influence in promoting anesthesia remain ambiguous. Given that VP is a crucial component in the indirect pathway of NAc^D2R^ neurons and recent research has demonstrated that activating the NAc^D1*R*^-VP pathway can facilitate awakening from general anesthesia,(Zhang et al., 2023) we propose the hypothesis that NAc^D2R^ neurons may modulate the process of general anesthesia through their interactions with VP. Further investigations are needed to elucidate the specific mechanisms and functional implications of the NAc^D2*R*^-VP pathway in the regulation of general anesthesia.

Our experimental manipulation of the NAc^D2*R*^-VP pathway through optogenetics yielded intriguing results. Activation of the NAc^D2*R*^-VP pathway led to a shortened induction time under sevoflurane anesthesia, accompanied by an increase in δ waves and a reduction in α, γ, and θ waves in the cortical EEG. Conversely, inhibition of the NAc^D2*R*^-VP pathway prolonged the induction time, reduced δ waves, and increased α and γ waves. These findings are in line with previous reports suggesting that the NAc^D2*R*^-VP pathway is involved in the regulation of sleep, (Luo Y. J. et al., 2018) where optogenetic activation of NAc indirect pathway neurons induces slow wave sleep, and chemogenetic inhibition suppresses slow wave sleep (Oishi and Lazarus, 2017). Our results further demonstrate that the NAc^D2*R*^-VP pathway not only plays a significant role in natural sleep processes but also contributes to the acceleration of anesthesia induction during general anesthesia.

Nevertheless, we have also observed that in comparison to the modulation of NAc^D2R^ neurons, which can impact the entirety of the anesthesia process, the regulation of the NAc^D2*R*^-VP pathway appears to have no significant effect on the anesthesia recovery process. This discrepancy might be attributed to the fact that the NAc^D2*R*^-VP pathway solely governs the anesthesia induction process. Current scientific literature supports the notion that numerous brain regions are exclusively dedicated to the anesthesia awakening process (Leung et al., 2014). For instance, in studies involving the ablation of VTA dopaminergic neurons, it was demonstrated that rats’ awakening time under propofol anesthesia was prolonged, while the anesthesia induction time remained unaffected (Zhou et al., 2015). The knockout of orexinergic neurons in mice can result in acquired narcolepsy and delay in anesthesia awakening, while the anesthesia induction time during sevoflurane anesthesia remains unchanged (Kelz et al., 2008). These findings imply that the mechanism governing general anesthesia induction and awakening may not be a reversible process solely controlled by a single neural network, and other neural circuits may also play a role in mediating the awakening delay caused by NAc^D2R^ neuron activation. For instance, Liu et al. (2017) identified a subgroup of GABAergic neurons (lhx6+) in the ventral zone (ZI) adjacent to the lateral hypothalamus (LH) that promote sleep. They conducted retrograde tracking experiments, confirming the monosynaptic projection of lhx6+ neurons from NAc to ZI (Liu et al., 2017). Based on the aforementioned theory and our own experimental results, we hypothesize that the NAc^D2*R*^-VP pathway is solely involved in regulating the induction process of sevoflurane anesthesia.

In conclusion, our experimental findings demonstrate the critical role of NAc^D2R^ neurons in the regulation of the anesthesia process. These neurons exert their influence on the anesthesia induction process through VP, highlighting this pathway as a key area for future investigations into the mechanisms underlying anesthesia-induced loss of consciousness. Furthermore, this study contributes a novel theoretical foundation to the understanding of general anesthesia mechanisms and offers a reliable basis for the development of rapid wake-up drugs for clinical anesthesia, potentially leading to reduced complications in clinical practice.

However, it is imperative to acknowledge the limitations of our study. Firstly, the fiber photometry technique exhibits certain constraints to some extent. For instance, this method is highly sensitive to photobleaching, particularly during prolonged recording sessions (exceeding 30 min). Furthermore, the signals we recorded emanate from a population response of D2R neurons that have been successfully transfected with GCaMP6s. Consequently, we cannot entirely rule out the possibility of non-transfected D2R neurons potentially becoming active during anesthesia. Additionally, the application of fiber photometry within the striatum may be susceptible to interference from other factors such as neuropil activity, and these factors should be considered within the scope of our study. Secondly, at the cellular level, the impact of sevoflurane on NAc^D2R^ neurons remains inadequately explored, necessitating further investigation through electrophysiological techniques. Thirdly, the precise neuronal identity of NAc^D2R^ projections to the ventral pallidum (VP) has yet to be definitively elucidated, necessitating further research and characterization to comprehensively understand the NAc^D2*R*^-VP pathway. Lastly, there is a possibility that NAc^D2R^ neurons may modulate general anesthesia through additional projection pathways, warranting further exploration. Addressing these limitations will contribute valuable insights, enhancing the depth and breadth of our comprehension in this field.

## Data availability statement

The original contributions presented in this study are included in this article/supplementary material, further inquiries can be directed to the corresponding authors.

## Ethics statement

The animal study was approved by the Animal Care and Use Committee of Zunyi Medical University. The study was conducted in accordance with the local legislation and institutional requirements.

## Author contributions

LN: Conceptualization, Data curation, Formal analysis, Investigation, Methodology, Software, Writing – original draft. MH: Investigation, Writing – review and editing. YW: Data curation, Writing – review and editing. KW: Data curation, Writing – review and editing. CY: Data curation, Writing – review and editing. YuZ: Writing – review and editing. JZ: Data curation, Writing – review and editing. XL: Data curation, Writing – review and editing. YiZ: Conceptualization, Writing – review and editing.

## References

[B1] Awal M. R. Austin D. Florman J. Alkema M. Gabel C. V. Connor C. W. (2018). Breakdown of neural function under isoflurane anesthesia: In Vivo, multineuronal imaging in Caenorhabditis elegans. *Anesthesiology* 129 733–743. 10.1097/ALN.0000000000002342 30004907 PMC6148381

[B2] Awal M. R. Wirak G. S. Gabel C. V. Connor C. W. (2020). Collapse of global neuronal states in caenorhabditis elegans under isoflurane anesthesia. *Anesthesiology* 133 133–144. 10.1097/ALN.0000000000003304 32282426 PMC7577177

[B3] Bao W. W. Xu W. Pan G. J. Wang T. X. Han Y. Qu W. M. (2021). Nucleus accumbens neurons expressing dopamine D1 receptors modulate states of consciousness in sevoflurane anesthesia. *Curr. Biol.* 31 1893–1902.e5. 10.1016/j.cub.2021.02.011 33705720

[B4] Bao W. Ding J. Jiang S. Yao Z. Qu W. Li W. (2023). Selective activation of NAc D1R-VP/LH circuits promotes reanimation from sevoflurane anesthesia in mice. *Anesth. Analg.* 137 87–97. 10.1213/ANE.0000000000006436 36944111

[B5] Bock R. Shin J. H. Kaplan A. R. Dobi A. Markey E. Kramer P. F. (2013). Strengthening the accumbal indirect pathway promotes resilience to compulsive cocaine use. *Nat. Neurosci.* 16 632–638. 10.1038/nn.3369 23542690 PMC3637872

[B6] Castro D. C. Bruchas M. R. (2019). A motivational and neuropeptidergic hub: Anatomical and functional diversity within the nucleus accumbens shell. *Neuron* 102 529–552. 10.1016/j.neuron.2019.03.003 31071288 PMC6528838

[B7] Fosque B. F. Sun Y. Dana H. Yang C. T. Ohyama T. Tadross M. R. (2015). Neural circuits. Labeling of active neural circuits in vivo with designed calcium integrators. *Science* 347 755–760. 10.1126/science.1260922 25678659

[B8] Franks N. P. (2008). General anaesthesia: From molecular targets to neuronal pathways of sleep and arousal. *Nat. Rev. Neurosci.* 9 370–386. 10.1038/nrn2372 18425091

[B9] Gerfen C. R. Surmeier D. J. (2011). Modulation of striatal projection systems by dopamine. *Annu. Rev. Neurosci.* 34 441–466. 10.1146/annurev-neuro-061010-113641 21469956 PMC3487690

[B10] Gretenkord S. Olthof B. M. J. Stylianou M. Rees A. Gartside S. E. LeBeau F. E. N. (2020). Electrical stimulation of the ventral tegmental area evokes sleep-like state transitions under urethane anaesthesia in the rat medial prefrontal cortex via dopamine D(1) -like receptors. *Eur. J. Neurosci.* 52 2915–2930. 10.1111/ejn.14665 31891427 PMC7497269

[B11] Gui H. Liu C. He H. Zhang J. Chen H. Zhang Y. (2021). Dopaminergic projections from the ventral tegmental area to the nucleus accumbens modulate sevoflurane anesthesia in mice. *Front. Cell Neurosci.* 15:671473. 10.3389/fncel.2021.671473 33994950 PMC8119636

[B12] Kelz M. B. Sun Y. Chen J. Cheng Meng Q. Moore J. T. Veasey S. C. (2008). An essential role for orexins in emergence from general anesthesia. *Proc. Natl. Acad. Sci. U.S.A.* 105 1309–1314. 10.1073/pnas.0707146105 18195361 PMC2234134

[B13] Kupchik Y. M. Brown R. M. Heinsbroek J. A. Lobo M. K. Schwartz D. J. Kalivas P. W. (2015). Coding the direct/indirect pathways by D1 and D2 receptors is not valid for accumbens projections. *Nat. Neurosci.* 18 1230–1232. 10.1038/nn.4068 26214370 PMC4551610

[B14] Leung L. S. Luo T. Ma J. Herrick I. (2014). Brain areas that influence general anesthesia. *Prog. Neurobiol.* 122 24–44. 10.1016/j.pneurobio.2014.08.001 25172271

[B15] Li Z. Chen Z. Fan G. Li A. Yuan J. Xu T. (2018). Cell-type-specific afferent innervation of the nucleus accumbens core and shell. *Front. Neuroanat.* 12:84. 10.3389/fnana.2018.00084 30459564 PMC6232828

[B16] Liu K. Kim J. Kim D. W. Stephanie Zhang Y. Bao H. Denaxa M. (2017). Corrigendum: Lhx6-positive GABA-releasing neurons of the zona incerta promote sleep. *Nature* 550:548. 10.1038/nature24274 28953871 PMC5695549

[B17] Lobo M. K. Nestler E. J. (2011). The striatal balancing act in drug addiction: Distinct roles of direct and indirect pathway medium spiny neurons. *Front. Neuroanat.* 5:41. 10.3389/fnana.2011.00041 21811439 PMC3140647

[B18] Luo T. Y. Cai S. Qin Z. X. Yang S. C. Shu Y. Liu C. X. (2020). Basal forebrain cholinergic activity modulates isoflurane and propofol anesthesia. *Front. Neurosci.* 14:559077. 10.3389/fnins.2020.559077 33192246 PMC7652994

[B19] Luo T. Yu S. Cai S. Zhang Y. Jiao Y. Yu T. (2018). Parabrachial neurons promote behavior and electroencephalographic arousal from general anesthesia. *Front. Mol. Neurosci.* 11:420. 10.3389/fnmol.2018.00420 30564094 PMC6288364

[B20] Luo Y. J. Li Y. D. Wang L. Yang S. R. Yuan X. S. Wang J. (2018). Nucleus accumbens controls wakefulness by a subpopulation of neurons expressing dopamine D(1) receptors. *Nat. Commun.* 9:1576. 10.1038/s41467-018-03889-3 29679009 PMC5910424

[B21] Mingote S. Amsellem A. Kempf A. Rayport S. Chuhma N. (2019). Dopamine-glutamate neuron projections to the nucleus accumbens medial shell and behavioral switching. *Neurochem. Int.* 129:104482. 10.1016/j.neuint.2019.104482 31170424 PMC6855309

[B22] Monti J. M. Jantos H. (2018). The effects of local microinjection of selective dopamine D1 and D2 receptor agonists and antagonists into the dorsal raphe nucleus on sleep and wakefulness in the rat. *Behav. Brain Res.* 339 11–18. 10.1016/j.bbr.2017.11.006 29137945

[B23] Oishi Y. Lazarus M. (2017). The control of sleep and wakefulness by mesolimbic dopamine systems. *Neurosci. Res.* 118 66–73. 10.1016/j.neures.2017.04.008 28434991

[B24] Oishi Y. Xu Q. Wang L. Zhang B. J. Takahashi K. Takata Y. (2017). Slow-wave sleep is controlled by a subset of nucleus accumbens core neurons in mice. *Nat. Commun.* 8:734. 10.1038/s41467-017-00781-4 28963505 PMC5622037

[B25] Pardo-Garcia T. R. Garcia-Keller C. Penaloza T. Richie C. T. Pickel J. Hope B. T. (2019). Ventral pallidum is the primary target for accumbens D1 projections driving cocaine seeking. *J. Neurosci.* 39 2041–2051. 10.1523/JNEUROSCI.2822-18.2018 30622165 PMC6507080

[B26] Root D. H. Melendez R. I. Zaborszky L. Napier T. C. (2015). The ventral pallidum: Subregion-specific functional anatomy and roles in motivated behaviors. *Prog. Neurobiol.* 130 29–70. 10.1016/j.pneurobio.2015.03.005 25857550 PMC4687907

[B27] Salgado S. Kaplitt M. G. (2015). The nucleus accumbens: A comprehensive review. *Stereotact. Funct. Neurosurg.* 93 75–93. 10.1159/000368279 25720819

[B28] Scofield M. D. Heinsbroek J. A. Gipson C. D. Kupchik Y. M. Spencer S. Smith A. C. (2016). The nucleus accumbens: Mechanisms of addiction across drug classes reflect the importance of glutamate homeostasis. *Pharmacol. Rev.* 68 816–871. 10.1124/pr.116.012484 27363441 PMC4931870

[B29] Tellez L. A. Perez I. O. Simon S. A. Gutierrez R. (2012). Transitions between sleep and feeding states in rat ventral striatum neurons. *J. Neurophysiol.* 108 1739–1751. 10.1152/jn.00394.2012 22745464 PMC3544947

[B30] van der Meij J. Martinez-Gonzalez D. Beckers G. J. L. Rattenborg N. C. (2019). Neurophysiology of avian sleep: Comparing natural sleep and isoflurane anesthesia. *Front. Neurosci.* 13:262. 10.3389/fnins.2019.00262 30983954 PMC6447711

[B31] Xu W. Wang L. Yuan X. S. Wang T. X. Li W. X. Qu W. M. (2020). Sevoflurane depresses neurons in the medial parabrachial nucleus by potentiating postsynaptic GABA(A) receptors and background potassium channels. *Neuropharmacology* 181:108249. 10.1016/j.neuropharm.2020.108249 32931816

[B32] Zhang J. Peng Y. Liu C. Zhang Y. Liang X. Yuan C. (2023). Dopamine D1-receptor-expressing pathway from the nucleus accumbens to ventral pallidum-mediated sevoflurane anesthesia in mice. *CNS Neurosci. Ther*. 29 3364–3377. 10.1111/cns.14267 37208941 PMC10580364

[B33] Zhang Y. Fu B. Liu C. Yu S. Luo T. Zhang L. (2019). Activation of noradrenergic terminals in the reticular thalamus delays arousal from propofol anesthesia in mice. *FASEB J.* 33 7252–7260. 10.1096/fj.201802164RR 30860868

[B34] Zhou X. Wang Y. Zhang C. Wang M. Zhang M. Yu L. (2015). The role of dopaminergic VTA neurons in general anesthesia. *PLoS One* 10:e0138187. 10.1371/journal.pone.0138187 26398236 PMC4580504

